# Efficacy and safety of Modified Tongxie Yaofang in diarrhea-predominant irritable bowel syndrome management: A meta-analysis of randomized, positive medicine-controlled trials

**DOI:** 10.1371/journal.pone.0192319

**Published:** 2018-02-06

**Authors:** Yun-kai Dai, Dan-yan Li, Yun-zhan Zhang, Meng-xin Huang, Yi-le Zhou, Jin-tong Ye, Qi Wang, Ling Hu

**Affiliations:** 1 Institute of Gastroenterology, Guangzhou University of Chinese Medicine, Guangzhou, Guangdong, China; 2 Institute of Clinical Pharmacology, Guangzhou University of Chinese Medicine, Guangzhou, Guangdong, China; Universita degli Studi di Napoli Federico II, ITALY

## Abstract

**Objective:**

To systematically evaluate the efficacy and safety of Modified Tongxie Yaofang (M-TXYF) for the treatment of diarrhea-predominant irritable bowel syndrome (IBS-D).

**Method:**

Electronic databases including PubMed, Springer Link, EMBASE, China National Knowledge Infrastructure (CNKI), Chinese Biomedical Literature (CBM), Wanfang, and Chinese Scientific Journals Database (VIP) were conducted from their inception through May 11, 2017 without language restrictions. Primary and secondary outcomes were estimated by 95% confidence intervals (CI). RevMan 5.3 and the Cochrane Collaboration’s risk of bias tool were analyzed for this meta-analysis.

**Results:**

Twenty-three literatures with a total of 1972 patients were included for the meta-analysis. The overall risk of bias evaluation was low. The pooled odds ratio showed that M-TXYF was significantly superior to routine pharmacotherapies (RP) in clinical therapeutic efficacy (OR 4.04, 95% CI 3.09, 5.27, *P* < 0.00001, therapeutic gain = 17.6%, number needed to treat (NNT) = 5.7). Moreover, compared with RP, M-TXYF showed that it can significantly reduce the scores of abdominal pain (standardized mean difference (SMD) -1.27; 95% CI -1.99, -0.56; *P* = 0.0005), abdominal distention (SMD -0.37; 95% CI -0.73, -0.01; *P* = 0.09), diarrhea (SMD -1.10; 95% CI -1.95, -0.25; *P* = 0.01), and frequency of defecation (SMD -1.42; 95% CI -2.19, -0.65; *P* = 0.0003). The differences of the adverse events between experiment and control groups had no statistical significance.

**Conclusion:**

This meta-analysis indicated that M-TXYF could be a promising Chinese herbal formula in treating IBS-D. However, considering the lack of higher quality of randomized controlled trials (RCTs), highly believable evidences should be required.

## Introduction

Irritable bowel syndrome (IBS) is a functional bowel disorder which is characterized by recurrent abdominal pain occurred at least 1 day per week in the last 3 months within a change in bowel habits [1–2]. Based on the predominant bowel habits according to Rome VI criteria [2], IBS is sub-classified into IBS with constipation (IBS-C), IBS with diarrhea (IBS-D), and mixed IBS (IBS-M). According to epidemiological investigation, approximately 5%-22% of general populations develop IBS [3–4], and up to 40% of patients have IBS-D [3]. This high prevalence results have significantly impaired the patients’ quality of life and medical costs [5].

Although tremendous efforts have been made to elaborate the cause of IBS, most routine pharmacotherapies (RP), including antidepressants, antispasmodic drugs, antidiarrheal drugs, and agents acting on 5-hydroxytryptamine (5-HT) receptor, have failed to achieve the desired clinical therapeutic efficacy. Furthermore, a numerous studies have verified that these drugs could potentially result in a risk of ischemic colitis and cardiovascular events [6].

With the development of traditional Chinese medicine (TCM), more and more IBS sufferers have turned to seek alternative treatments, particularly Chinese herbal medicine. Tongxie Yaofang (TXYF), an ancient formula in treating IBS-D with liver-qi stagnation and spleen deficiency [7], is often modified with different Chinese herbal additions based on syndrome differentiation. To date, two publications have reported TCM in treating IBS. One was a systematic review of TXYF for IBS [8]. In this review, because of poor quality in most included trials, the definitive conclusions have not been drawn. Moreover, sub-classification of IBS was also left out of consideration. The other study was a meta-analysis of Shugan Jianpi Zhixie therapy for IBS-D [9]. Although this sub-classification was taken into consideration, placebos were adopted in control groups. Considering the medical ethics, the use of them was irresponsible for patients. Recently, several well-designed clinical studies evaluating Modified TXYF (M-TXYF) for IBS-D have been issued [10–32]. Therefore, in order to acquire more precise and reliable results, we conducted a meta-analysis to estimate the efficacy and safety of M-TXYF for IBS-D.

## Methods

### Search strategy

To identify relevant literatures, electronic search was comprehensively conducted for publications in the following 6 electronic databases: PubMed, Springer Link, EMBASE, China National Knowledge Infrastructure (CNKI), Wanfang, and Chinese Scientific Journals Database (VIP). The general wording of the search terms were individually used or in combination: “Tongxie Yaofang”, “traditional Chinese medicine”, “Chinese medicinal herb”, “traditional Chinese herbal formula”, “herbs”, “irritable bowel syndrome”, “IBS”, “irritable colitis”, “functional bowel disease”, “allergic colitis”, “colon allergy”, “randomized controlled trials (RCTs)”, “clinical trial”. The retrieval time was from their inception through May 11, 2017 without language restrictions. A detailed search strategy for each of the databases could be found in S1 Search Strategy. Omissive relevant literatures were supplemented by manual search.

### Study selection

Studies with the following eligibility criteria were conducted for quantitative analysis: (1) All the included trials are randomized controlled trials (RCTs) in humans. (2) IBS-D is definitively diagnosed on the basis of Rome II, III, or VI. (3) All participants are adults. (4) Experiment groups should present the efficacy of M-TXYF in comparison with RP. (5) Pregnant women and patients with malignant tumor or severe cardiovascular diseases are excluded.

### Data extraction and quality assessment

Extracting data was independently conducted by two researchers. The contents of extracted data were composed of the following items: first author, the year of publishing, western criteria, TCM criteria, study population, ages, sample sizes, intervention, treatment sessions, outcome measurements, clinical therapeutic efficacy, follow-up, and side effects. Evaluation of methodological quality, supplemented by Jadad score [33], was performed based on the Cochrane Collaboration’s risk of bias tool [34]. Literature with a Jadad score below 3 was deemed inferior quality article, whereas literature with a score above 3 was regarded as superior one. Based on the contents of Jadad score, the quality of literatures were divided into three different grades as follows [33]:

A = Low risk of bias for literatures with a score of 5
B = Moderate risk of bias for literatures with a score of 1–4
C = High risk of bias for literatures with a score of 0

Disputes emerging between two investigators were settled after discussion or by another one.

### Data synthesis and analysis

Review Manager 5.3 was used for the meta-analysis. Odds ratio (OR) and 95% confidence intervals (CI) were estimated for dichotomous data. Standardized mean difference (SMD) and 95% CI were calculated for continuous variable data. A statistical test for heterogeneity was conducted with *χ*^*2*^ test and inconsistency index statistic (*I*^*2*^) [35]. If significant heterogeneity existed (*I*^*2*^ >50% or *P* <0.05), the pooled OR was evaluated by using a random effect model. Otherwise, a fixed effect model was used.

A sensitivity analysis was performed to investigate underlying sources of heterogeneity. Meanwhile, it can also estimate the robustness of emerging results through sequentially omitting one trial. Therapeutic gain reciprocal was described as the number needed to treat (NNT). Publication bias was qualitatively detected by Begg’s funnel plot.

## Results

**Fig 1:**
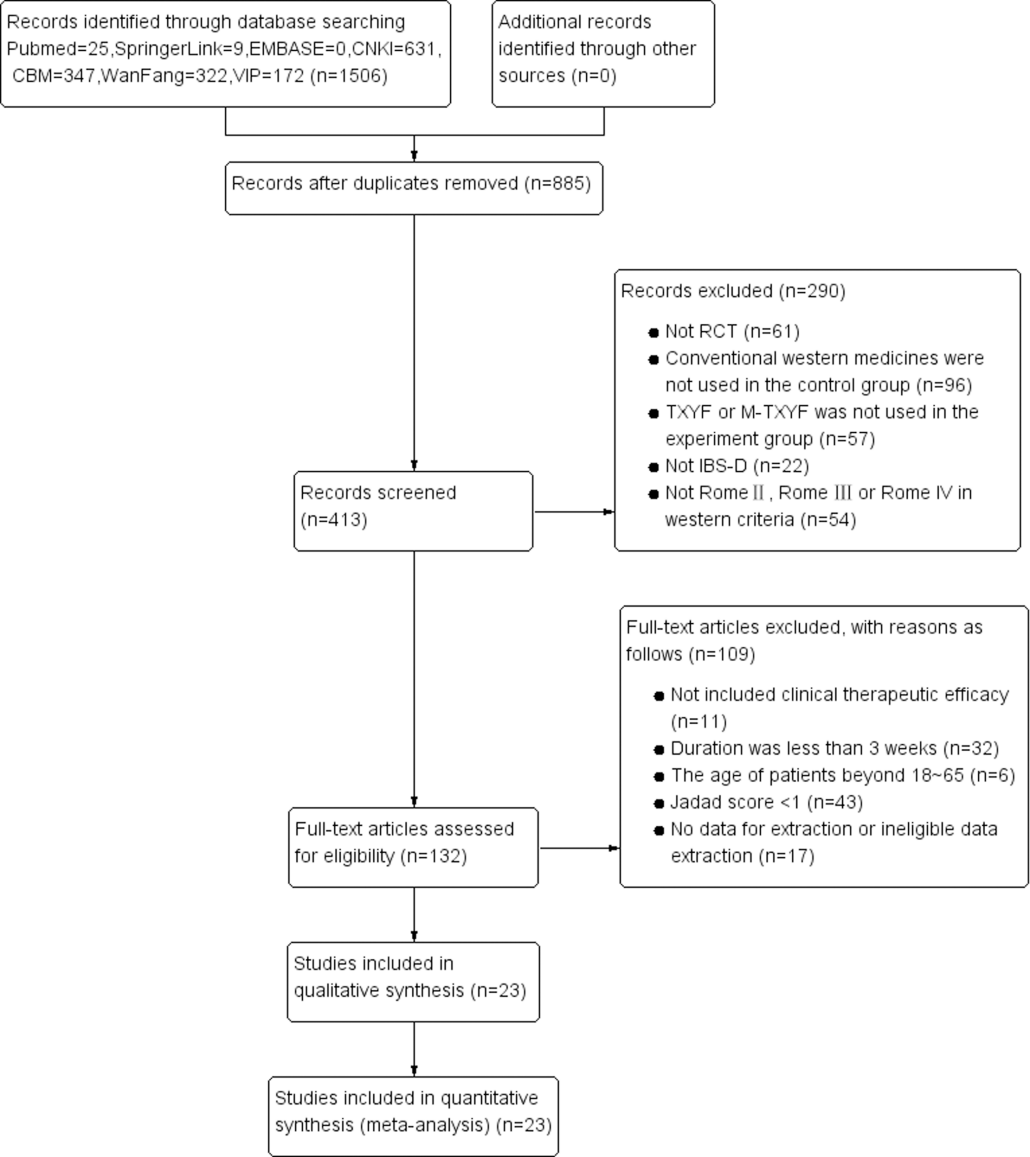
Flow chart of the process for literature retrieval.

A total of 1,506 relevant studies were identified based on computer search. Both investigators agreed on the results of inclusion criteria and showed detailed screening process for study identification and selection in Fig 1. Finally, twenty-three RCTs met the inclusion criteria for the meta-analysis [10–32]. Literature characteristics are described in Table 1.

**Table 1:** Characteristics of included literature.

Included studies (First Author, Year)	Western criteria	TCM criteria	Study population	Ages (years old)	Sample Size			Intervention		Treatment sessions	Outcome measurements	Side effects
					E(M/F)	C(M/F)		E	C			
An et al. 2017 (10)	RomeIII	N.D	Single center	E: 34.8±3.8 C: 34.2±3.6	32(19/13)	32(21/11)		M-TXYF	Montmorillonite powder + PBT	4 weeks	A+B+E+F+O+R+Z+AA	no
Chen et al. 2016 (11)	RomeIII	LSASD	Single center	E: 43.2±18.3 C: 42.1±17.5	30(12/18)	30(13/17)		M-TXYF	PBT	4 weeks	A+B+C+D+H+R	N.D
Ge et al. 2016 (12)	RomeIII	LSASD	Single center	E: 36.8±7.2 C: 37.4±6.7	62(30/32)	62(28/34)		M-TXYF	PBT	4 weeks	A+B+D+E+F+O+Y	E: 2 cases C: 10 cases
Ma et al. 2016 (13)	RomeIII	DBLAS	Single center	18–65	23(10/13)	23(9/14)		M-TXYF	PBT	4 weeks	A+B+C+D	no
Qian et al. 2016 (14)	RomeIII	LSAAS	Single center	32.13±4.22	**E**/**C**: 30/30 (M: 20 F: 40)			M-TXYF	PBT	3 weeks	A+B+C+F	no
Li et al. 2015 (15)	RomeIII	LSASD	Single center	E: 42.27±2.31 C: 41.40±2.36	30(13/17)	30(12/18)		M-TXYF + CBT	PBT	4 weeks	A+B+C	N.D
Li 2015 (16)	RomeII	LSASD	Single center	18–48	**E**/**C:** 42/42 (M: 58 F: 26)			M-TXYF	PBT	8 weeks	A+B+E+F+G+H+I	N.D
Peng et al. 2014 (17)	RomeIII	LSASD	Single center	N.D	**E**/**C**: 27/23			M-TXYF	PBT	4 weeks	A+P+U	no
Wen et al. 2014 (18)	RomeIII	LSASD	Single center	E: 41.7±11.6 C: 42.4±12.3	42(16/26)		42(14/28)	M-TXYF	PBT	4 weeks	A+B+E+F	no
Chen et al. 2014 (19)	RomeIII	LSAAS	Single center	E: 38.48±11.93 C: 38.35±11.75	58(38/20)		58(32/26)	M-TXYF	PBT	4 weeks	A+I+J+K+Q	C: 4 cases
Zhang et al. 2014 (20)	RomeIII	N.D	N.D	E: 47.13±8.27 C: 46.58±8.44	44(24/20)		44(21/23)	M-TXYF	Trimebutine maleate + Montmorillonite powder	4 weeks	A+B+E+N+O+R	no
Tu 2013 (21)	RomeIII	N.D	Single center	E: 39.8±9.6 C: 39.5±8.6	56(38/18)		48(33/15)	M-TXYF	Bacillus licheniformis + PBT	4 weeks	A+AB	N.D
Wang et al. 2013 (22)	RomeIII	LSASD	Single center	E: 27±4.5 C: 29±5.1	48(28/20)		50(24/26)	M-TXYF	PBT	4 weeks	A+B+C+E+L+M	N.D
Wang et al. 2012 (23)	RomeIII	LSASD	Single center	E: 42.5±12.5 C: 43.2±11.7	45(16/29)		45(14/31)	M-TXYF	Montmorillonite powder	4 weeks	A+B+C+G+AC+AD+AE+AF	no
Tao et al. 2012 (24)	RomeIII	LSASD	Single center	E: 40.85±11.62 C: 41.14±12.08	68(40/28)		64(37/27)	M-TXYF	PBT	4 weeks	A+J+V+W	N.D
Xu et al. 2011 (25)	RomeIII	LSASD	Single center	E: Mean: 43 C: Mean: 44	52(30/22)		52(31/21)	M-TXYF	PBT	4 weeks	A	N.D
Zhang 2010 (26)	RomeIII	LSASD	Single center	E: 38.5±2.27 C: 36.6±20.7	40(16/24)		40(14/26)	M-TXYF	Loperamide	4 weeks	A	N.D
Zhang et al. 2009 (27)	RomeIII	LSASD	Single center	E: 39.98±12.24 C: 37.67±10.96	53(30/23)		54(35/19)	M-TXYF	PBT	4 weeks	A+B+D+E+F+I	N.D
Liang et al. 2009 (28)	RomeIII	DBLAS	Multi- center	38.65±4.93	20(7/13)		20(9/11)	M-TXYF	PBT	4 weeks	A+B+D+E+F+N+O	no
Pan et al. 2009 (29)	RomeIII	N.D	Single center	E: 39.2±13.4 C: 37.5±15.6	80(33/47)		40(17/23)	M-TXYF	Miyarisam	4 weeks	A+B+D+F+AG	N.D
Gao et al. 2009 (30)	RomeII	LSASD	Single center	N.D	**E**/**C:** 78/26			M-TXYF	Glutamine compound enteric capsule	3 weeks	A+B+E+F+X	no
Fang 2008 (31)	RomeIII	LSASD	Single center	25–69	40(18/22)		40(19/21)	M-TXYF	PBT + Live Combined bifidobacterium + lactobacillus and enterococcus Powder	12 weeks	A	E: 1case C: 6cases
Cai et al. 2006 (32)	RomeII	LSASD	Single center	E: 47.32±12.81 C: 47.48±11.60	60(31/29)		31(20/11)	M-TXYF	PBT	8 weeks	A+B+D+E+F+R+S+T	N.D

Annotation: A: clinical therapeutic efficacy; B: abdominal pain score; C: diarrhea score; D: abdominal distention score; E: frequency of defecation score; F: property of stool score; G: borborygmus score; H: the level of gastrointestinal hormones; I: IBS-QOL score; J: IBS-BSS score; K: IBS-DSQ; L: sleeping quality score; M: diet condition score; N: poor stool output score; O: mucous stool score; P: gastrointestinal hormones; Q: TCM-PES; R: defecation’s condition score; S: satisfaction of defecation; T: disturbance of life; U: TCM symptom score; V: TCM symptom therapeutic effect; W: QOL(SF-36) scale; X: abdominal discomfort; Y: the level of serum brain gut peptide; Z: SAS (self-rating anxiety scale) scores; AA: SDS (self-rating depression scale) scores; AB: recurrence rate; AC: tension score; AD: fullness and discomfort in chest and hypochondrium score; AE: belching score; AF: poor appetite score; AG: mental condition score

LSASD: Liver-qi stagnation and spleen deficiency; LSAAS: Liver-qi stagnation and attacking spleen; DBLAS: Disharmony between liver and spleen; PBT: Pinaverium bromide tablets; N.D: not described; **E:** Experiment group; **C:** Control group.

**Fig 2:**
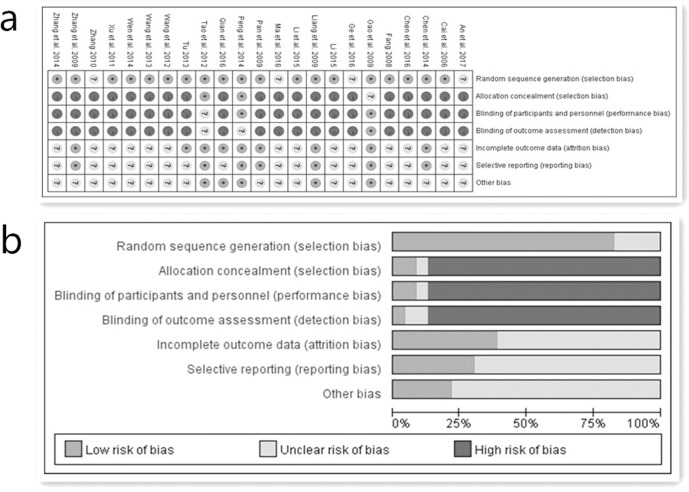
(a) Risk of bias summary. (b) Risk of bias graph.

A description of the assessment for methodological quality is shown in Table 2. In this systematic review, nineteen trials described how patients were randomly assigned into the experiment groups and the control groups (fifteen used a random number table [12, 15–17, 20–27, 29, 31, 32], two used a block randomization [18, 33], one used a random grouping table [19], and one used a stratified block randomization [30]). However, the remaining four studies did not report the specific randomization technique [11, 13, 14, 28]. In addition, only the Peng et al. study used a single-blind [18]. Only the Gao et al. study used a double-blind and double-dummy [31]. As for allocation concealment, only the Peng et al. study and the Tao et al. study used lightproof envelopes [18, 25]. In addition, eight trials reported follow-up after treatment [15, 18, 20, 22, 25, 28–30]. Although withdrawals and dropouts were mentioned in seven studies [18, 20, 25, 28–31], only the Peng et al. study [17] conducted intention-to-treat (ITT) analysis. The Chen et al. study [19] used full analysis sets (FAS). The Gao et al. study conducted full analysis set (FAS), per-protocol population set (PPS) and safe set (SS) [30]. However, the remaining studies did not conduct intention-to-treat (ITT) analysis for the cases of dropouts [10–16, 18, 20–29, 31, 32]. In a word, because of lacking specific information, it cannot be determined whether implementations were adequately performed in the process of random sequence generation, blinding or allocation concealment. Therefore, the validity of this review in Fig 2 could be regarded as high risk.

**Table 2:** Assessment of methodological quality of the qualified studies.

Included Studies	Baseline	Randomization	Blinding	Allocation concealment	Follow-up	Withdrawals and dropouts	Jadad score	Quality of Literature
An et al. 2017 (10)	Comparability	MBND	N.D	N.D	N.D	N.D	1	B
Chen et al. 2016 (11)	Comparability	Random number table	N.D	N.D	N.D	N.D	2	B
Ge et al. 2016 (12)	Comparability	MBND	N.D	N.D	N.D	N.D	1	B
Ma et al. 2016 (13)	Comparability	MBND	N.D	N.D	N.D	N.D	1	B
Qian et al. 2016 (14)	Comparability	Random number table	N.D	N.D	1 month	N.D	2	B
Li et al. 2015 (15)	Comparability	Random number table	N.D	N.D	N.D	N.D	2	B
Li 2015 (16)	Comparability	Random number table	N.D	N.D	N.D	N.D	2	B
Peng et al. 2014 (17)	Comparability	Block randomization	Single-blind	Lightproof envelope	MBND	E: 1 case C: 2 cases	3	B
Wen et al. 2014 (18)	Comparability	Random grouping table	N.D	N.D	N.D	N.D	2	B
Chen et al. 2014 (19)	Comparability	Random number table	N.D	N.D	8 weeks	E: 3 cases C: 3 cases	3	B
Zhang et al. 2014 (20)	Comparability	Random number table based on the proportion of 1:1	N.D	N.D	N.D	N.D	2	B
Tu 2013 (21)	Comparability	Random number table	N.D	N.D	3 months after treatment course;1 year for curative cases	N.D	2	B
Wang et al. 2013 (22)	Comparability	Random number table	N.D	N.D	N.D	N.D	2	B
Wang et al. 2012 (23)	Comparability	Random table	N.D	N.D	N.D	N.D	2	B
Tao et al. 2012 (24)	Comparability	Random number table based on the proportion of 1:1	N.D	Lightproof envelope	3rd and 6th month after treatment course	E: 2 cases C: 6 cases	3	B
Xu et al. 2011 (25)	Comparability	Random number table	N.D	N.D	N.D	N.D	2	B
Zhang 2010 (26)	Comparability	Random number table	N.D	N.D	N.D	N.D	2	B
Zhang et al. 2009 (27)	Comparability	Randomization based on the proportion of 1:1	N.D	N.D	after 2nd and 4th weeks during the treatment course;1st and 3rd months after treatment course	E: 2 cases C: 1 case	3	B
Liang et al. 2009 (28)	Comparability	Random number table based on the proportion of 1:1	N.D	N.D	3 months	no	2	B
Pan et al. 2009 (29)	Comparability	Stratified block randomization	N.D	N.D	MBND	E: 3 cases	3	B
Gao et al. 2009 (30)	Comparability	Random number table based on the proportion of 3:1	Double-blind double-dummy	N.D	N.D	E group:4 cases C group:4 cases	5	A
Fang 2008 (31)	Comparability	Random number table	N.D	N.D	N.D	N.D	2	B
Cai et al. 2006 (32)	Comparability	Block randomization based on the proportion of 2:1	N.D	N.D	N.D	N.D	2	B

Annotation: N.D: not described; MBND: mentioned but not described; E: Experiment group; C: Control group.

### Primary outcome measurement: clinical therapeutic efficacy

#### Comparison of clinical therapeutic efficacy

A total of 23 included studies reported the efficacy, which included 1972 patients where 1052 and 920 participants were respectively assigned to M-TXYF and RP [10–32]. Based on the “Guiding Principle for Clinical Research of New Drugs of TCM” [36], clinical therapeutic efficacy was calculated in the comprehensive efficacy index (CEI): CEI(%) = (the numbers of patients whose clinical symptom improved after intervention divide total numbers of patients) × 100%.

#### Subgroup analysis

**Fig 3:**
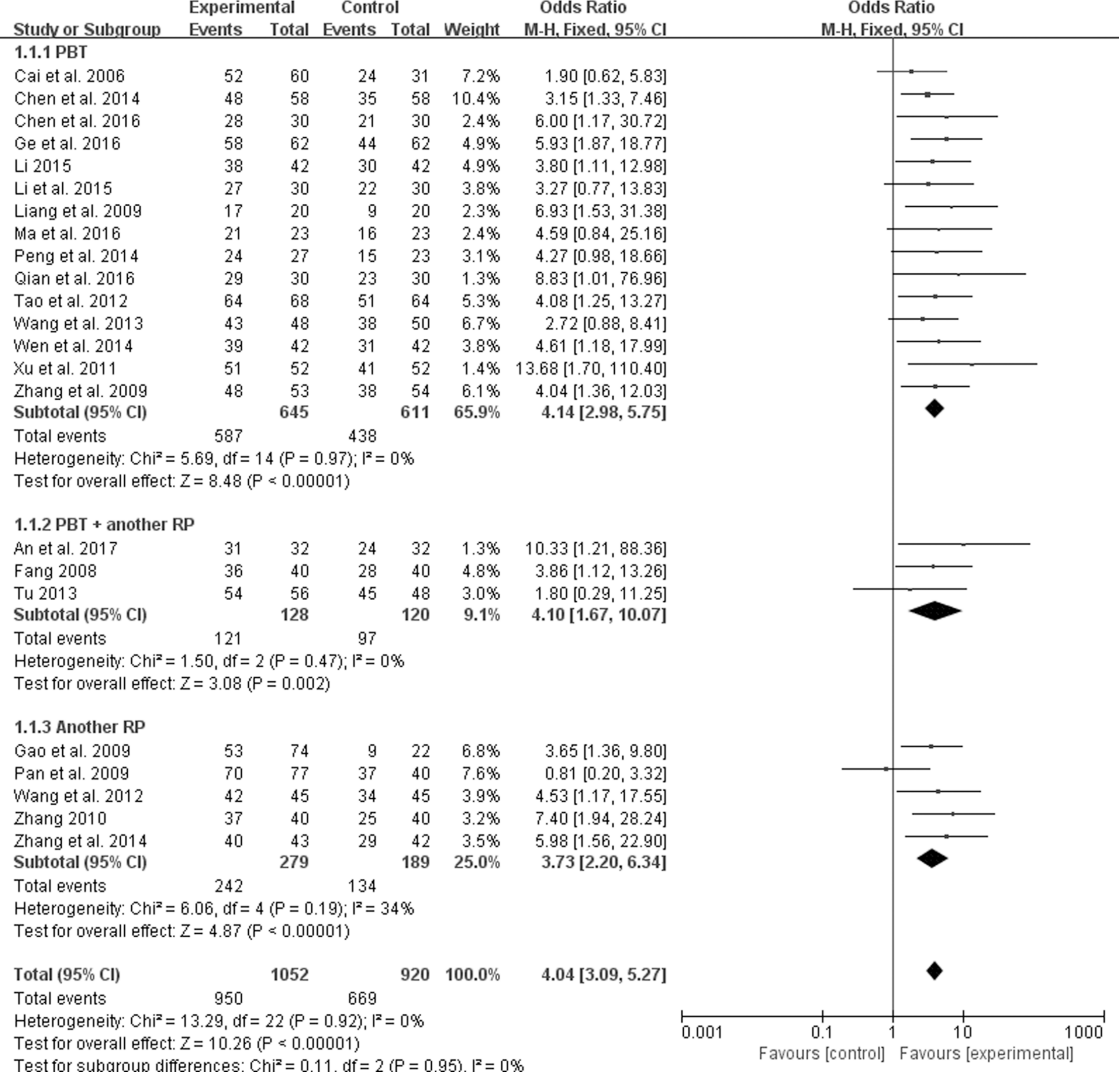
Forest plot of clinical therapeutic efficacy.

**Fig 4:**
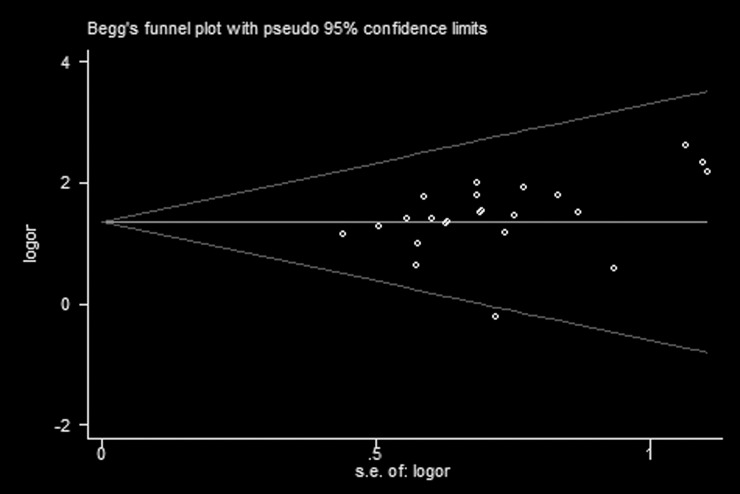
Funnel plot analysis of clinical therapeutic efficacy.

Because of variability in evaluating point of clinical therapeutic efficacy, we conducted subgroup analysis among studies using different interventions of Pinaverium bromide tablets (PBT), PBT + another RP (including Montmorillonite powder, Trimebutine maleate, Bacillus licheniformis, Loperamide, Miyarisam, Glutamine compound enteric capsule, Live combined bifidobacterium, Lactobacillus and enterococcus), and another RP. Compared with the control groups, the experiment groups were positive effects on the improvement of clinical symptoms for PBT (OR 4.14; 95% CI 2.98, 5.75; *P* < 0.00001) in fifteen trials [11–19, 22, 24, 25, 27, 28, 32], PBT + another RP (OR 4.14; 95% CI 1.67, 10.07; *P* = 0.002) in three trials [10, 21, 31], another RP (OR 3.73; 95% CI 2.20, 6.34; *P* < 0.00001) in five trials [20, 23, 26, 29, 30], and an overall efficacy (OR 4.04; 95% CI 3.09, 5.27; *P* < 0.00001) in Fig 3. In addition, the results in Table 3 showed that 90.3% of treatment group participants had a clinical effectiveness, which was superior to 72.7% of control group participants (therapeutic gain = 17.6% with NNT = 5.7). Potential publication bias (Begg’s test, *P* = 0.009) was identified by observing the asymmetrical plot in Fig 4.

**Table 3:** Clinical therapeutic efficacy, M-TXYF vs. RP.

Study	Effective rate, % (efficacy/N)		Therapeutic gain, %	NNT	OR (95% CI)
	M-TXYF	Positive medicine			
An et al. 2017 (10)	96.9 (31/32)	75.0 (24/32)	21.9	4.6	10.33 (1.21, 88.36)
Chen et al. 2016 (11)	93.3 (28/30)	70.0 (21/30)	23.3	4.3	6.00 (1.17, 30.72)
Ge et al. 2016 (12)	93.5 (58/62)	71.0 (44/62)	22.5	4.4	5.93 (1.87, 18.77)
Ma et al. 2016 (13)	91.3 (21/23)	69.6 (16/23)	21.7	4.6	4.59 (0.84, 25.16)
Qian et al. 2016 (14)	96.7 (29/30)	76.7 (23/30)	20.0	5.0	8.83 (1.01, 76.96)
Li et al. 2015 (15)	90.0 (27/30)	73.3 (22/30)	16.7	6.0	3.27 (0.77, 13.83)
Li 2015 (16)	90.5 (38/42)	71.4 (30/42)	19.1	5.2	3.80 (1.11, 12.98)
Peng et al. 2014 (17)	88.9 (24/27)	65.2 (15/23)	23.7	4.2	4.27 (0.98, 18.66)
Wen et al. 2014 (18)	92.9 (39/42)	73.8 (31/42)	19.1	5.2	4.61 (1.18, 17.99)
Chen et al. 2014 (19)	82.8 (48/58)	77.6 (35/58)	5.2	19.2	3.15 (1.33, 7.46)
Zhang et al. 2014 (20)	90.7 (39/43)	69.0 (29/42)	21.7	4.6	5.98 (1.56, 22.90)
Tu 2013 (21)	96.4 (54/56)	93.8 (45/48)	2.6	38.5	1.80 (0.29, 11.25)
Wang et al. 2013 (22)	89.5 (43/48)	76.0 (38/50)	13.5	7.4	2.72 (0.88, 8.41)
Wang et al. 2012 (23)	93.3 (42/45)	75.6 (34/45)	17.7	5.6	4.53 (1.17, 17.55)
Tao et al. 2012 (24)	94.1 (64/68)	79.7 (51/64)	14.4	6.9	4.08 (1.25, 13.27)
Xu et al. 2011 (25)	98.1 (51/52)	79.0 (41/52)	19.1	5.2	13.68 (1.70, 110.40)
Zhang 2010 (26)	93.0 (37/40)	63.0 (25/40)	30.0	3.3	7.40 (1.94, 28.24)
Zhang et al. 2009 (27)	90.6 (48/53)	70.4 (38/54)	20.2	5.0	4.04 (1.36, 12.03)
Liang et al. 2009 (28)	85.0 (17/20)	45.0 (9/20)	40.0	2.5	6.93 (1.53, 31.38)
Pan et al. 2009 (29)	90.9 (70/77)	92.5 (37/40)	-1.6	-62.5	0.81 (0.20, 3.32)
Gao et al. 2009 (30)	71.6 (53/74)	40.9 (9/22)	30.7	3.3	3.65 (1.36, 9.80)
Fang 2008 (31)	90.0 (36/40)	70.0 (28/40)	20.0	5.0	3.86 (1.12, 13.26)
Cai et al. 2006 (32)	86.7 (52/60)	77.4 (24/31)	9.3	10.8	1.90 (0.62, 5.83)
Pooled OR	90.3 (950/1052)	72.7 (669/920)	17.6	5.7	4.04 (3.09, 5.27)

NNT, number needed to treat; OR, odds ratio.

### Secondary outcome measurements

#### Abdominal pain score

**Fig 5:**
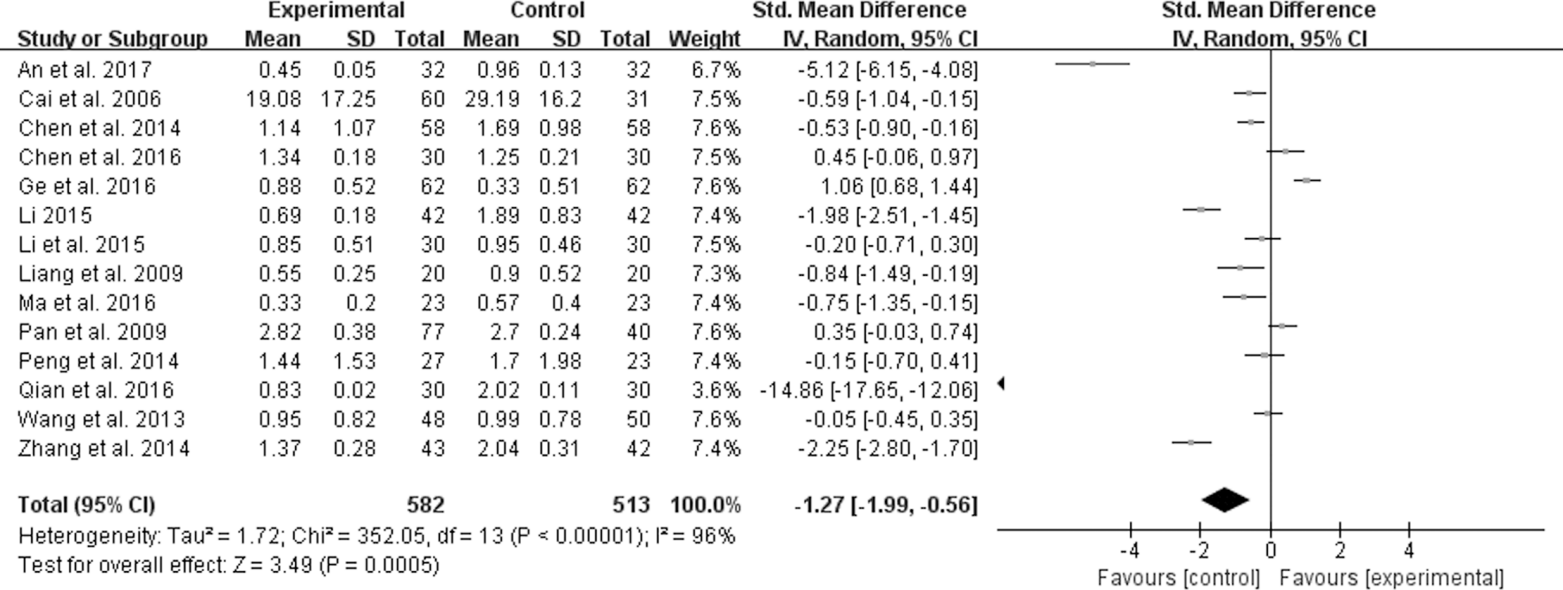
Forest plot of abdominal pain.

Among the included studies, fourteen reported the improvement of abdominal pain [10–17, 19, 20, 22, 28, 29, 32]. However, owing to the existence of substantial heterogeneity (*χ*^*2*^ = 352.05, *P* < 0.00001, *I*^*2*^ = 96%), a random effect model was applied to estimating pooled effect sizes. Furthermore, the forest plot in Fig 5 showed that the reduction of abdominal pain score in experiment groups was more remarkable than that in control groups (SMD -1.27; 95% CI -1.99, -0.56; *P* = 0.0005).

#### Abdominal distention score

**Fig 6:**
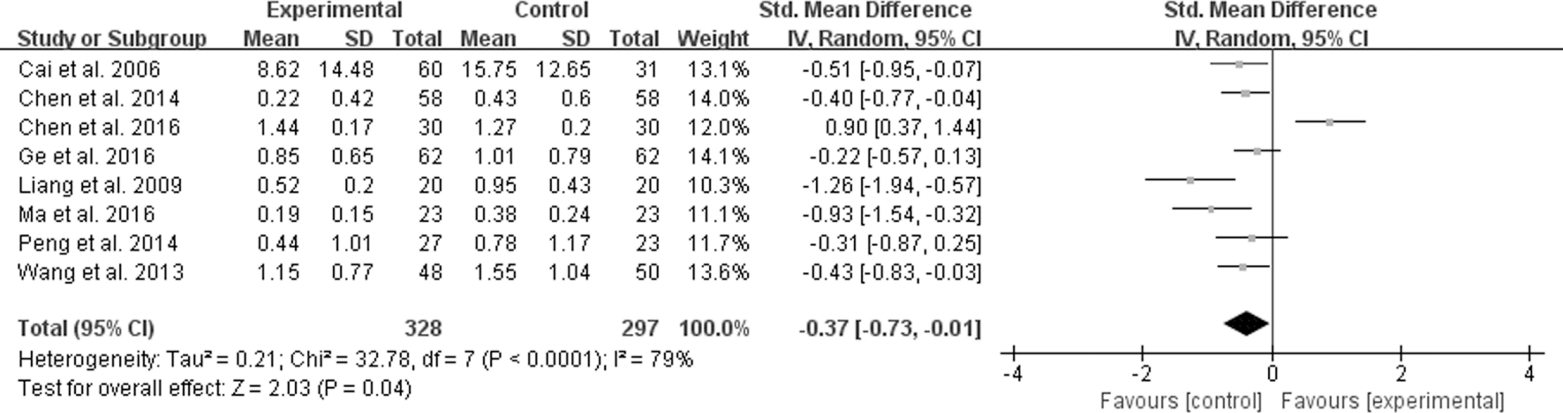
Forest plot of abdominal distention.

Eight studies reported abdominal distention score [11–13, 17, 19, 22, 28, 32]. A random effect model was conducted because of significant heterogeneity (*χ*^*2*^ = 32.78, *P* < 0.0001, *I*^*2*^ = 79%). However, the result of meta-analysis in Fig 6 showed that M-TXYF had no statistically significant differences in alleviation for abdominal distention compared with RP (SMD -0.37; 95% CI -0.73, -0.01; *P* = 0.09).

#### Diarrhea score

**Fig 7:**
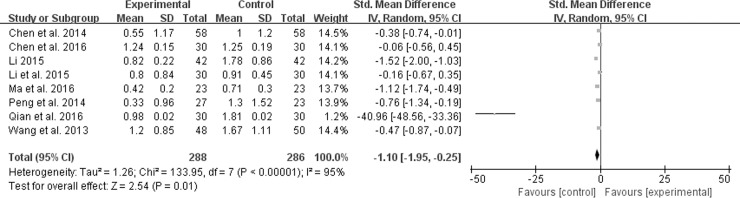
Forest plot of diarrhea.

Of all the included studies, eight reported the diarrhea score [11, 13–17, 19, 22]. A model of random effect was conducted to estimate pooled effect sizes because of significant heterogeneity (*χ*^*2*^ = 133.95, *P* < 0.00001, *I*^*2*^ = 95%). Treatment groups in Fig 7 showed more significant improvement of diarrhea than control groups (SMD -1.10; 95% CI -1.95, -0.25; *P* = 0.01).

#### Frequency of defecation score

**Fig 8:**
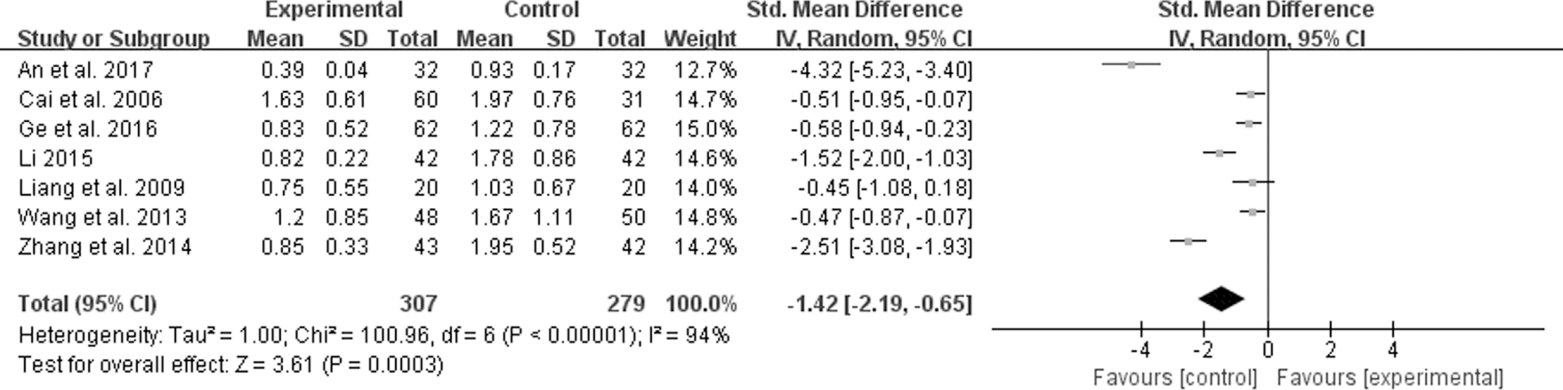
Forest plot of frequency of defecation.

In the included trials, seven described the situation of defecation’s frequency [10, 12, 16, 20, 22, 28, 32]. Random-effect meta-analysis in Fig 8 indicated that M-TXYF had positive effects on frequency of defecation changes (*χ*^*2*^ = 100.96, *P* < 0.00001, *I*^*2*^ = 94%) (SMD -1.42; 95% CI -2.19, -0.65; *P* = 0.0003).

#### BSS score

Two included trials applied bowel symptom severity scale (BSS) to estimate the severity of IBS-D symptoms [19, 24]. Considering the lack of enough numbers of literatures, the overall BSS score were only qualitatively analyzed in order to avoid inaccurate and unreliable outcomes. But in improving bowel symptom severity, the experiment groups had potentially superior to the control groups.

#### QOL scale

Four qualified literatures reported health-related quality of life (QOL) [16, 19, 24, 27]. One used the Chinese version of the short form 36 (SF-36) which was validated in Hong Kong [24]. Another one used QOL scale whose contents contained psychological and physical dimensions, and social adaptability [16]. The remaining two studies used IBS-QOL which was authorized by Patrick et al. [19, 27]. Although the four literatures elaborated different ingredients of QOL scale, they all showed that M-TXYF could significantly improve health-related QOL.

### Safety evaluation

Twelve studies assessed the safety of M-TXYF in the course of treatment [10, 12–14, 17–20, 23, 28, 30, 31]. One trial reported 2 cases of nausea occurred in the experiment group and 10 cases of adverse reactions (4 for nausea, 4 for local skin rash, and 2 for abdominal discomfort) occurred in the control group [12]. In addition, although one trial reported 4 cases of side effects (2 for constipation, 1 for nausea, and 1 for dry mouth) occurred in the control group [19], all of these side effects spontaneously disappeared after the treatment session. Besides, the Fang study reported 1 case of nausea occurred in the experiment group and 6 cases of adverse effects (2 for constipation, 2 for nausea, and 2 for skin rash) occurred in the control group [31]. But these side effects did not have impact on the experimental process. In addition, nine trials had no adverse reactions during M-TXYF treatment [10, 13, 14, 17, 18, 20, 23, 28, 30].

## Discussion

This meta-analysis concluded that M-TXYF could exhibit better clinical therapeutic efficacy than RP. And compared with control groups, M-TXYF showed significant reduction of scores in secondary outcome measurements. In addition, the Li et al. study [15] reported cognitive-behavior treatment based on M-TXYF, which aimed at propagandizing cognitive education to patients. However, all of the included studies indicated that publication bias potentially existed. In addition, although there were three trials [12, 19, 31] appeared mild adverse effects in treatment course, these did not exert negative influence on the treatment of IBS-D.

Results of this meta-analysis have validated that RP could effectively improve the symptoms of this disorder [37–39]. However, M-TXYF may be more superior to RP in the reduction of symptom scores, with therapeutic gains over these RP of 17.6% and NNT = 5.7. The reported NNT offered by otilonium was 7 and by combining otilonium with alverine/simethicone was 8 [40]. In other words, M-TXYF is more likely to show better clinical therapeutic efficacy than otilonium or combined with alverine/simethicone for IBS-D. Therefore, multi-center, randomized, and double-blind trials which are to assess the efficacy and safety of M-TXYF for IBS-D should be worthy of further research.

IBS pathophysiology remains inadequately explained [39, 40]. Numerous mechanisms indicated that IBS was associated with altered colonic motility, abnormal colonic flora, visceral hypersensitivity, inflammation, and psychological factors [41–44]. A substantial number of modern pharmacological studies have verified the clinical effectiveness of TXYF for IBS-D. Experimental investigation has elucidated that TXYF can effectively alleviate abdominal pain and diarrhea symptoms of IBS-D rats, whose mechanisms may be achieved through adjusting the colonic mucosa ion channels, changing colon dynamics of the colon and further affecting the secretion and absorption of colonic mucosa [45, 46]. In addition, some research data have shown that TXYF can significantly neutralize emotions and reduce visceral sensory hypersensitivity by regulating brain-gut interaction and the expression of cellular oncogene fos (c-fos) mRNA, 5-HT_4_ mRNA, somatostatin (SS), and plasma vasoactive intestinal peptide (VIP) in gastrointestinal hormones in human beings or rats with IBS-D [47–50]. To acquire more concrete and detailed mechanisms, further studies should be conducted in vitro and in vivo of TXYF or M-TXYF for IBS-D.

The methodological quality of included trials was general moderate. Furthermore, the potential risk of bias in our study was possibly derived from three fields. First, blinding was not adopted for most included trials, making participants realized which treatment they were taken and resulting in the emergence of performance and detection biases. Second, only two studies conducted allocation concealment [17, 25]. Therefore, investigators and patients were easily conscious of which group they were allocated to, thereby causing inevitable selection bias. Finally, as for withdrawals and dropouts, only three studies conducted intention-to-treat (ITT) analysis [17, 19, 30], which may generate half-baked outcome data. In addition, based on equivalence trial principles [51] in the Tao et al. study [25], there were eight patients excluded, but the concrete reasons why these patients dropped out were not elaborated. Besides, there were three patients withdrew in the Zhang et al. study [27]. One was on business after receiving treatment. Another thought it was inconveniences for him to decoct and take Chinese medicine. The third one was examined for colorectal polyps after entering the group. In a word, most of these studies lacked ITT analysis, leading to incomplete outcome data and increasing the risk of attrition bias.

Limitations of this meta-analysis were as follows. First, the populations of included studies were Chinese, not involving foreigners. This geographically limited distribution could also result in publication and cultural biases in IBS diagnosis. Moreover, due to the diversities of diet structure and lifestyle among them, it was hard to popularize the efficacy of M-TXYF for IBS-D throughout the world. In addition, most of the qualified studies were single centers and small sample sizes. Therefore, the efficacy of M-TXYF applied to future multi-center and large-scale trials should await further proof. Next, although all Chinese herbal formulas in experiment group were based on TXYF, all formulas included different additional herb(s). Therefore, because of a lack of standardization, these differences may result in different patients taking different ingredient decoctions, thus affecting effectiveness. Moreover, these Chinese medicinal herbs were purely natural. Meanwhile, variation in the herbs themselves, including source and preparation, might be the source of heterogeneous. Finally, IBS, as a chronic recurrent disease, should take adequate treatment durations and follow-up periods into consideration. However, treatment courses in the most studies reviewed here were three to four weeks, which was too short to assess the long-term efficacy and safety of M-TXYF for IBS-D. Although eight included trials reported the follow-up visits [14, 17, 19, 21, 24, 27–29], all of them only evaluated short- or medium-term efficacy of M-TXYF for IBS-D. Furthermore, because of the characteristics of IBS, long-term follow-up visits played a significant role in this chronic disease. Therefore, sufficient time for follow-up visits should be essential to accurately evaluate the efficacy and safety of M-TXYF.

## Conclusions

The meta-analysis indicates that M-TXYF could be superior to RP in treating IBS-D. Meanwhile, it can also potentially reduce the scores of abdominal pain, abdominal distention, diarrhea and frequency of defecation. Although M-TXYF is safe in short- and medium-term trials, there is no strong evidence to verify its efficacy in treating IBS for long term. Therefore, recommendations of specific M-TXYF for IBS-D cannot be made at present and these results should be interpreted with great caution. Furthermore, highly believable evidences are required, as well as well-designed, large-scale, multi-center, randomized controlled and double-blinded trials.

## Supporting information

S1 PRISMA Checklist (DOC)

S1 Search Strategy (DOC)

## References

[1] Stanghellini V, Chan FK, Hasler WL, Malagelada JR, Suzuki H, Tack J, et al Gastroduodenal Disorders. Gastroenterology. 2016; 150(6):1380–1392. doi: 10.1053/j.gastro.2016.02.011 27147122 10.1053/j.gastro.2016.02.011

[2] Lacy BE, Mearin F, Chang L, Chey WD, Lembo AJ, Simren M, et al Bowel Disorders. Gastroenterology. 2016; 150(6):1393–1407. 10.1053/j.gastro.2016.02.031 27144627

[3] Lovell RM, Ford AC. Global prevalence of, and risk factors for, irritable bowel syndrome: a meta-analysis. Clin Gastroenterol Hepatol. 2012; 10(7): 712–721. doi: 10.1016/j.cgh.2012.02.029 22426087 10.1016/j.cgh.2012.02.029

[4] Grundmann O, Yoon SL. Irritable bowel syndrome: epidemiology, diagnosis and treatment: an update for health-care practitioners. J Gastroenterol Hepatol. 2010; 25(4): 691–699. doi: 10.1111/j.1440-1746.2009.06120.x 20074154 10.1111/j.1440-1746.2009.06120.x

[5] Simrén M, Svedlund J, Posserud I, Björnsson ES, Abrahamsson H. Health-related quality of life in patients attending a gastroenterology outpatient clinic: functional disorders versus organic diseases. Clin Gastroenterol Hepatol. 2006; 4(2): 187–195. 16469679 10.1016/s1542-3565(05)00981-x

[6] Brandt LJ, Chey WD, Foxx-Orenstein AE, Schiller LR, Schoenfeld PS, Spiegel BM, et al An evidence-based position statement on the management of irritable bowel syndrome. Am J Gastroenterol. 2009; 104(Suppl 1): S1–35. 10.1038/ajg.2008.122 19521341

[7] Sung JJ, Leung WK, Ching JY, Lao L, Zhang G, Wu JC, et al Agreements among traditional Chinese medicine practitioners in the diagnosis and treatment of irritable bowel syndrome. Aliment Pharmacol Ther. 2004; 20(10): 1205–1210. doi: 10.1111/j.1365-2036.2004.02242.x 15569124 10.1111/j.1365-2036.2004.02242.x

[8] Bian Z, Wu T, Liu L, Miao J, Wong H, Song L, et al Effectiveness of the Chinese herbal formula TongXieYaoFang for irritable bowel syndrome: a systematic review. J Altern Complement Med. 2006; 12(4): 401–407. doi: 10.1089/acm.2006.12.401 16722791 10.1089/acm.2006.12.401

[9] Xiao Y, Liu Y, Huang S, Sun X, Tang Y, Cheng J, et al The Efficacy of Shugan Jianpi Zhixie Therapy for Diarrhea-Predominant Irritable Bowel Syndrome: A Meta-Analysis of Randomized, Double-Blind, Placebo-Controlled Trials. PLoS ONE. 2015; 10(4): e0122397 doi: 10.1371/journal.pone.0122397 25853241 10.1371/journal.pone.0122397 PMC4390216

[10] An LF, Li HH, Chang CQ, Han TT, Duan ZY, Wang ML. Clinical curative effect of Jiawei Tongxie Yaofang on irritable bowel syndrome (diarrhea type). Journal of Shanxi College of Traditional Chinese Medicine. 2017; 18(2): 68–71.

[11] Chen JL, Chen JF, Deng JM, Han YB. Influence of Jiawei Tongxie decoction to recipe on liver stagnation and spleen deficiency type of irritable bowel syndrome patients with small intestinal mucosal 5-HT and its receptor mRNA expression. Chin J Integr Trad West Med Dig. 2016; 24(6): 442–445.

[12] Ge F, Ji Y, Sun ZG, Zhu SL, Dai HF. Exploration on Mechanism of Treating Diarrhea Predominant Irritable Bowel Syndrome with Jianchang Yihao Formula. J Nanjing Univ Tradit Chin Med. 2016; 32(3): 213–216.

[13] Ma DY, Peng LL. Clinical Observation on Sini powder combined with Modified Tongxie Yaofang in treating Diarrhea-Predominant Irritable Bowel Syndrome. SHANXI J OF TCM. 2016; 32(1): 16–17.

[14] Qian X, Zhang J, Zhang YL. The observation of efficacy and safety on Modified Tongxie Yaofang in treating Diarrhea-Predominant Irritable Bowel Syndrome. Zhejiang Journal of Integrated Traditional Chinese and Western Medicine. 2016; 26(10): 941–943.

[15] Li SJ, Xu SY, Lin RJ. Analysis of intestinal Changshu Granule combined with CBT in treating irritable bowel syndrome. Journal of LiaoNing University of Traditional Chinese Medicine. 2015; 17(9): 85–87.

[16] Li K. The clinical research of Pain laxative side addition and subtraction of irritable bowel syndrome quality of life in patients with liver and spleen deficiency diarrhea. Lab Med Clin. 2015; 12(12): 1707–1709.

[17] Peng MZ, Wang SY, Li X, Chen S, Li W, Zhang L, et al Clinical Study on Liver-Dispersing and Spleen-Strengthening Treatment on Diarrhea Predominant Irritable Bowel Syndrome. World Chinese Medicine. 2014; 9(12): 1595–1598.

[18] Wen PY, Lai Y. Forty-Two Cases with Diarrhea-Predominant Irritable Bowel Syndrome of Liver-qi Stagnation and Spleen Deficiency Treated by SiJunZi Decoction combined with Modified TongXieYaoFang. Fujian Journal of Traditional Chinese Medicine. 2014; 45(1): 30–31.

[19] Chen MX, Chen JX, Xia L, Fu R, Lu Z. Treating irritable bowel syndrome with diarrhea patiens by yigan fupi decoction: a randomized controlled trial. Chin J Integr Trad West Med. 2014; 34(6): 656–660. 25046944

[20] Zhang LN, Liu ZP, Liao ZF. Treatment for 44 Cases of Diarrhea-Irritable Bowel Syndrome by *XieGan JianPi BuShen Tang*. Western Journal of Traditional Chinese Medicine. 2014; 27(9): 120–122.

[21] Tu Y. A Randomized Controlled Study for Tongxieyaofang Treating Diarrhea Type Irritable Bowel Syndrome. Journal of Practical Traditional Chinese Internal Medicine. 2013; 27(1): 41–42.

[22] Wang YX, Huo YL, Wang W. Clinical Observation on Modified Tongxie Yaofang in treating IBS with Diarrhea of Liver Depression and Spleen Deficiency Syndrome. HENAN Traditional Chinese Medicine. 2013; 33(1): 67–69.

[23] Wang HF, Zhang J, Zhang J, Wang JQ. 45 Cases of Irritable Bowel Syndrome (Diarrhea Type) Treated with the Modified Tongxie Yaofang. World Journal of Integrated Traditional and Western Medicine. 2012; 7(5): 425–427.

[24] Tao L, Zhang SS, Xiao Y, Li W, Shen C, Wu B. Influence on the quality of life for patients with diarrhea-predominant irritable bowel treated by spleen-strengthening and liver-dispersing therapy. Beijing Journal of Traditional Chinese Medicine. 2012; 31(6): 437–440.

[25] Xu MH, Niu KM, Niu XD. Clinical Observation on Tongxie Yaofang with different Chinese herbal additions in treating 104 cases with Diarrhea-Predominant Irritable Bowel Syndrome. Nei Mongol Journal of Traditional Chinese Medicine. 2011; 3021(21): 38–40.

[26] Zhang GS. Clinical Observation on Modified Tongxie Yaofang in treating Diarrhea-Predominant Irritable Bowel Syndrome. Modern Journal of Integrated Traditional Chinese and Western Medicine. 2010; 19(28): 3612–3613.

[27] Zhang SS, Xu WJ, Chen Z, Chen J. Short-term and Medium-term Clinical Effect of Liver Dispersing with Spleen Strengthening on Irritable Bowel Syndrome Dominated by Diarrhea. Journal of Capital Medical University. 2009; 30(4): 436–440.

[28] Liang ZF, Chen RH, Xu YS, Chen QX, Dong ML. Tiaohe Ganpi Hexin Decoction in treatment of irritable bowel syndrome with diarrhea: a randomized controlled trial. J Chin Integr Med. 2009; 7(9): 819–822. 10.3736/jcim20090904 19747435

[29] Pan F, Zhang T, Zhang YH, Xu JJ, Chen FM. Effect of Tongxie Yaofang Granule in Treating Diarrhea-Predominate Irritable Bowel Syndrome. Chin J Integr Med. 2009; 15(3): 216–219. doi: 10.1007/s11655-009-0216-7 19568715 10.1007/s11655-009-0216-7

[30] Gao WY, Lin YF, Chen SQ, Lu YP, Yang Z, Gong Y, et al Effects of Changjishu soft elastic capsule in treatment of diarrhea-predominant irritable bowel patients with liver-qi stagnation and spleen deficiency syndrome: a randomized double-blinded controlled trial. Journal of Chinese Integrative Medicine. 2009; 7(3): 212–217. 19284948 10.3736/jcim20090303

[31] Fang P. Clinical Effect of Jiaweitongxie Prescription in Treating Irritable Bowel Syndrome. Zhejiang Journal of Integrated Traditional Chinese and Western Medicine

[32] Cai G, Lei YX, Zheng SH, Li YM. Clinical Observation on Chang Ji Tai in treating sixty cases with diarrhea-predominant irritable bowel syndrome. Jiangxi Journal of Traditional Chinese Medicine. 2006; 37(5): 20–21.

[33] Jadad AR, Moore RA, Carroll D, Jenkinson C, Reynolds DJ, Gavaghan DJ, et al Assessing the quality of reports of randomized clinical trials: is blinding necessary? Control Clin Trials. 1996 2;17(1):1–12. 8721797 10.1016/0197-2456(95)00134-4

[34] Savović J, Weeks L, Sterne JA, Turner L, Altman DG, Moher D, et al Evaluation of the Cochrane Collaboration’s tool for assessing the risk of bias in randomized trials: focus groups, online survey, proposed recommendations and their implementation. Syst Rev. 2014 4 15;3:37 doi: 10.1186/2046-4053-3-37 24731537 10.1186/2046-4053-3-37 PMC4022341

[35] Higgins JP, Thompson SG, Deeks JJ, Altman DG. Measuring inconsistency in meta-analyses. BMJ. 2003; 327(7414): 557–560. doi: 10.1136/bmj.327.7414.557 12958120 10.1136/bmj.327.7414.557 PMC192859

[36] Zheng XY, ed. Guiding principle of clinical research on new drugs of traditional Chinese medicine (Trial). Beijing: China Medical Science and Technology Press 2002; 139–143.

[37] Poynard T, Regimbeau C, Benhamou Y. Meta-analysis of smooth muscle relaxants in the treatment of irritable bowel syndrome. Aliment Pharmacol Ther. 2001; 15(3): 355–361. 11207510 10.1046/j.1365-2036.2001.00937.x

[38] Lόpez-Alvarenga JC, Sobrino-Cossío S, Remes-Troche JM, Chiu-Ugalde J, Vargas-Romero JA, Schmulson M. Polar vectors as a method for evaluating the effectiveness of irritable bowel syndrome treatments: An analysis with pinaverium bromide 100mg plus simethicone 300mg po bid. Rev Gastroenterol Mex. 2013; 78(1): 21–27. doi: 10.1016/j.rgmx.2012.10.003 23375823 10.1016/j.rgmx.2012.10.003

[39] Zheng L, Lai Y, Lu W, Li B, Fan H, Yan Z, et al Pinaverium reduces symptoms of irritable bowel syndrome in a multicenter, randomized, controlled trial. Clin Gastroenterol Hepatol. 2015; 13(7): 1285–1292. doi: 10.1016/j.cgh.2015.01.015 25632806 10.1016/j.cgh.2015.01.015

[40] Martínez-Vázquez MA, Vázquez-Elizondo G, González-González JA, Gutiérrez-Udave R, Maldonado-Garza HJ, Bosques-Padilla FJ. Effect of antispasmodic agents, alone or in combination, in the treatment of Irritable Bowel Syndrome: Systematic review and meta-analysis. Rev Gastroenterol Mex. 2012; 77(2): 82–90. doi: 10.1016/j.rgmx.2012.04.002 22672854 10.1016/j.rgmx.2012.04.002

[41] Törnblom H, Lindberg G, Nyberg B, Veress B. Full-thickness biopsy of the jejunum reveals inflammation and enteric neuropathy in irritable bowel syndrome. Gastroenterology. 2002; 123(6): 1972–1979. doi: 10.1053/gast.2002.37059 12454854 10.1053/gast.2002.37059

[42] Rapps N, van Oudenhove L, Enck P, Aziz Q. Brain imaging of visceral functions in healthy volunteers and IBS patients. J Psychosom Res. 2008; 64(6): 599–604. doi: 10.1016/j.jpsychores.2008.02.018 18501260 10.1016/j.jpsychores.2008.02.018

[43] Ruepert L, Quartero AO, de Wit NJ, van der Heijden GJ, Rubin G, Muris JW. Bulking agents, antispasmodics and antidepressants for the treatment of irritable bowel syndrome. Cochrane Database Syst Rev. 2001; (8). 10.1002/14651858.CD003460.pub3 PMC8745618 21833945

[44] Deechakawan W, Heitkemper MM, Cain KC, Burr RL, Jarrett ME. Anxiety, depression, and catecholamine levels after self-management intervention in irritable bowel syndrome. Gastroenterology nursing: the official journal of the Society of Gastroenterology Nurses and Associates. 2014; 37(1): 24–32. 24476829 10.1097/SGA.0000000000000017

[45] Yang C, Zhang SS, Xiong Y, Wang ZF. Effects of Tong-Xie-Yao-Fang Formula on Colonic Movement Index and Contraction Frequency of D-IBS Rats. World Chinese Medicine. 2015; 10(5): 675–678.

[46] Zhang SS, Yang C. Effects of TongXie-YaoFang Formula on serotonin and chromogranin a related transport mechanism of ion channels in colonic mucosa in diarrhea-predominant irritable bowel syndrome. Journal of Capital Medical University. 2015; 36(4): 645–648.

[47] Zhao YP, Wang FY, Yang JQ, Su M, Tang XD. Research progress in the pathogenesis of irritable bowel syndrome based on abnormal brain-gut interaction. Natl Med J China. 2015; 95(8): 637–640.

[48] Li DH, Bai X, Xie XL, Geng JG. Mechanisms for Tongxie Yaofang to Treat Irritable Bowel Syndrome from Point of View of Interaction between Brain and Intestine. Chinese Journal of Experimental Traditional Medicine Formulae. 2010; 16(12): 118–121. doi: 10.13422/j.cnki.syfjx.2010.12.013

[49] Xu HJ, Ten C, Qian YQ, Liu HH, Wang YJ, Chai JY, et al Effect of Tongxieyaofang on Vasoactive Intestinal Polypeptide and VPAC1 in Rats of Diarrhea-predominant Irritable Bowel Syndrome. Chinese archives of traditional Chinese medicine. 2012; 30(2): 268–270.

[50] Wang JW, Ye HY, Zhao WJ, Yin Y, Zhao XY, Xu FH, et al Effect of Tongxie Yaofang on expression of 5-HT4 receptor mRNA and c-fos mRNA on colon tissue of IBS visceral hypersensitivity rats. China Journal of Traditional Chinese Medicine and Pharmacy. 2014; 29(4): 1070–1075.

[51] Zhu Q, Liang WX. Principles to specify equivalence margin in clinical trials. Chin J Clin Pharmacol Ther. 2005; 10(8): 957–960

