# Supplementary material for: Efficacy and safety of Modified Tongxie Yaofang in diarrhea-predominant irritable bowel syndrome management: A meta-analysis of randomized, positive medicine-controlled trials
Source: PLoS One. 2018 Feb 6;13(2):e0192319. doi: 10.1371/journal.pone.0192319 (PMC5800650; doi:10.1371/journal.pone.0192319)
Supplement: S1 Search Strategy — (DOC) [file pone.0192319.s002.doc]

**Search strategy for each of the databases**

**PubMed:**

#1 irritable bowel syndrome [SH]

#2 IBS [SH]

#3 irritable colitis [SH]

#4 exp functional bowel disease [SH]

#5 exp allergic colitis [SH]

#6 exp colon allergy [SH]

#7 #1 or #2 or #3 or #4 or #5 or #6

#8 Tongxie Yaofang [SH]

#9 exp traditional Chinese medicine [SH]

#10 exp Chinese medicinal herb [SH]

#11 exp traditional Chinese herbal formula [SH]

#12 exp herbs [SH]

#13 #8 or #9 or #10 or #11 or #12

#14 randomized controlled trial [PT]

#15 controlled clinical trial [PT]

#16 randomized [TIAB]

#17 #14 or #15 or #16

#18 #7 and #13 and #17

Annotation: Mesh Subheadings [SH] Publication Type [PT] Title/Abstract [TIAB]

**EMBASE:**

1 (irritable bowel syndrome or IBS or irritable colitis).af.

2 exp functional bowel disease/ or exp allergic colitis/ or exp colon allergy/

3 1 or 2

4 (Tongxie Yaofang).af.

5 exp traditional Chinese medicine/ or exp Chinese medicinal herb/ or exp traditional Chinese herbal formula/ or exp herbs/

6 4 or 5

7 (random* OR factorial* OR crossover*).af.

8 exp crossover-procedure/ or exp double-blind procedure/ or exp randomized controlled trial/ or single-blind procedure/

9 7 or 8

10 3 and 6 and 9

**Springer Link:**

#1 Tongxie Yaofang OR traditional Chinese medicine OR Chinese medicinal herb OR traditional Chinese herbal formula OR herbs

#2 irritable bowel syndrome OR IBS OR irritable colitis OR functional bowel disease OR allergic colitis OR colon allergy

#3 randomized controlled trial OR controlled clinical trial OR randomized

#4 #1 AND #2 AND #3

**CNKI** (China National Knowledge Infrastructure):

#1 Tongxie Yaofang

#2 irritable bowel syndrome OR IBS

#3 randomized controlled trial OR controlled clinical trial OR randomized

#4 #1 AND #2 AND #3

**Wanfang:**

#1 Tongxie Yaofang

#2 irritable bowel syndrome OR IBS

#3 randomized controlled trial OR controlled clinical trial OR randomized

#4 #1 AND #2 AND #3

**VIP** (Chinese Scientific Journals Database):

#1 Tongxie Yaofang

#2 irritable bowel syndrome OR IBS

#3 randomized controlled trial OR controlled clinical trial OR randomized

#4 #1 AND #2 AND #3
